# 

**Published:** 1847-06

**Authors:** 


### WESTERN JOURNAL

### OF

### MEDICINE AND SURGERY.

### EDITORS.

DANIEL DRAKE, M. D.,

PROFESSOR OF PATHOLOGY AND THE PRACTICE OF MEDICINE IN THE UNIVERSITY OF LOUISVILLE.

LUNSFORD P. YANDELL, M. D.,

PROFESSOR OF CHEMISTRY AND PHARMACY IN THE SAME.

THOMAS W. COLESCOTT, M. D.

### INDEX TO VOL. VII.

[NEW SERIES.]

Abscess of the jaws, 276.
Afterpains, 440, 447.
Agassiz on the sea-serpent, 522.
Age, effects of, 144.
Alcoholic liquids, 154.
Alkalies in hooping cough, 155.
Amenorrhea, 502.
Ames on scarlatina and measles, 118
Ammonia in glaciers, 239.
Aneurism, 169, 359.
Ani prolapsus, 220.
Anthrax, 102, 224.
Aphthæ, 431.
Apoplectic chill, 245.
Army of occupation, diseases of, 185, 537.
Arsenic in furuncles, 112.
Artery temporal, anomalous, 220.
Asthma, ether in, 524.
Asylum lunatic, 178.
Aura epileptica, 309.

Baldness, causes of, 438.
Bedford's introductory, 146.
Bennet on the uterus, 341.
Bladder, rupture of, 267.
Blind, Kentucky Institution for, 361
Bostock Dr. death of, 360.
Brain, compression of, 518.
Brain, concussion of, 18.
Bromide of potassium, 455.

Cachexia, iron in, 61.
Cæsarian operation, 63.
Caldwell's introductory, 90, 144.
Camphor, poisoning by, 459.
Cancer, 163.
Cancer of the bones, 317, 369.
Carbonic acid, poisoning by, 459.
Carbuncle, 102.
Chapman on medical reform, 534.
Chemistry organic, 173.
Chest, Latham on, 407.
Children, diseases of, 47, 505.
Child born alive at four months, 359
Chill apoplectic, 245.
Chlorosis, tannate of iron in, 6.
Chlorosis, sulphate of iron in, 317.
Cholera Asiatic, 166, 525.
Chorea cured by strychnine, 222.
Clamart, 100.
Clinical lecture, New York, 498.
Coley on children, 47.
Combustion spontaneous, 334.
Concealed delivery, 74.
Condie on children, 505.
Convalescence, management in, 443
Convention medical, 260, 364, 540.
Copaiba as an injection, 106.
Coral islands, Dumas on, 214.
Croton oil frictions in phthisis, 328.
Croup, 83, 402.

Darrach's introductory, 275.
Dawson on eclampsia, 277.
Deglutition excited by cold water, 438
Deposits in human body, 266.
Diarrhea, 108, 170.
Dickson's introductory, 181,
Digestion of alcohol, 154.
Digestive organs, diseases of, 525.
Diseases of Tennessee troops, 185.
Draderick's excision of lower jaw, 12
Drake Dr.'s letter, 547.
Dropsy, 237.
Dumas, 96, 214,

Eclampsia parturientium, 277.

Electricity in disease, 163.
Epistaxis, 359.
Ergot in afterpains, 440.
Erysipelas, 106, 113, 309.
Ether in asthma and hooping c. 524.
Ether in surgical operations, 67, 331, 429, 500.
Ether in the passage of gravel, 453.

Fee on quinine, 296.
Female, characteristics of, 271.
Fever in Alabama, 128.
Fever intermittent, 201.
Fever, iron with quinine in, 84.
Fevers miasmatic, 355.
Fever typhoid, 112, 359.
Fistula vesico-vaginal, 480.
Fistulous opening in the lung, 497.
Fodder, green and dry, 174.
Forbes on hydropathy, 38.
Foster's case of spontaneous combustion, 334.
Fracture of the skull, 113.
Fungous tumors, 110.
Furuncles, arsenic in, 112.

Geological researches, 149.
Gold in syphilis, 308.
Gonorrhea, 78.
Gonorrhea, tinct. mur. ferri in, 92.
Gordon's case of hydatids, 9.
Grayson Springs, 142.
Greenly on intermittent fever, 201.
Growth of plants, light on, 175.
Greenly's case of fracture, 113.
Gregory's case of poisoning, 333.
Gun cotton, 7, 521.
Gunpowder, 535.

Harris on fever, 128.
Heart, a coagulum in, 323.
Hemorrhage from the gums, 247.
Hernia, 153, 321.
Hernia crural, 461.
Homœopathy in Germany, 260,
Hooping cough, alkalies in, 155.
Hooping cough, ether in, 524.
Horsford on grains, 239.
Hospitals in Paris, 232.
Hydatids uterine, 9.
Hydatids of the liver, 315.
Hydrocele, 230, 466.
Hydropathy, 38.

Iodine injections in hydrocele, 466.
Iodide of potash in rheumatism, 112
Iron in cachexia, 61.
Iron, sulphate in chlorosis, 317.
Iron, tannate in chlorosis, 6.
Iron with quinine in fevers, 84.

Jackson's introductory, 91.
Jobert on nit. argent, 309.
Jones's ophthalmic surgery, 346.

Kentucky Institution for the blind, 361.
Knee joint, disease of, 248.

Latham on the chest, 407.
Lethean vapor, 276, 349.
Light on the growth of plants, 175.
Lime in diarrhea, 170.
Lime oxalate of, 437.
Lithotomy and lithotrity, 62, 354.
Louis's case of consumption, 226.

Marable's case, amenorrhea, etc. 502
Materia Medica by Royle, 335.
Maxillary bone, excision of, 12.
McClellan Dr. Geo. death of, 543.
McCoy's case of inversio uteri, 16.
Measles, 118.
Medical convention, 260, 364, 540.
" " Ohio, 547.
Medical Soc. N. Y. Transac. 518.
Medico-legal case, 404.
Meigs's cases of double vagina, 81.
Mercury oxide of in vomiting, 167.
Mesmerism in surgery, 6.
Monesia, 231.
Morgue, 233.
Mortality in army and navy, 168.
Moxa, 6.

Nelaton on cancer of the bones, 317, 369.
Nervous diseases, 520.
Neuralgia periodical, 17.
Nitrate of silver, 309, 317.
Nitric acid in Bright's disease, 108.

Œsophagus obliteration of, 158.
Ointment mercurial in carbuncle, 224
Orchitis, 1.
Orfila, 94.
Osborne on mur. of iron in gon. 92.

Osborne's cases of dropsy, 237.

Parker Prof. clinic, 498.
Pere la Chaise, 234.
Perineum, infiltration of urine in, 468
Peritonitis, 497.
Peyer's glands in fevers, 534.
Phillips on scrofula, 20.
Philosophy in sport, 427
Phlebitis, 380.
Phrenology, 263.
Phthisis, Louis' case of, 226.
Plagiarism, 523.
Pneumonia, 196, 440, 442, 503.
Poisoning by datura stramonium,333
Polypus uteri. 198.
Prison discipline, 544.

Quackery, profits of, 519.
Quinine in rheumatism, 105.
" its modus operandi, 296.
" how to disguise its taste, 314
" action of on spleen, 358.
" poisonous action, 450.

Revere Prof. death of, 543.
Rheumatism, nit. argent. in, 307.
" quinine in, 105.
Robards on disease of army, 185.
Roux on fungous tumors, 110, 223, 318.
Royle's Materia Medica, 335.
Rupture of the bladder, 267.
" umbilical cord, 529.
" vagina, 532.

Sanguinaria Canadensis, 397,
Scarlatina, 118.
Scrofula, 20.
Scruggs's cases, 196.
Sea-sickness, 330.
Sea-serpent, structure of, 522.
Skull, fracture of, 113.
Smallpox, 229, 329.
Somnambulism, 156.
Spleen, quinine on, 358.
Statistics, army and obstetrical, 80.
" miasmatic fevers, 355.
Strother and Mattingly's case, 404.
Strychnia in chorea, 222.
Strychnia in tetanus, 501.
Students medical, in U. States and France, 84.
Sugar in urine, test for, 466.
Syphilis tertiary, 501.
Taylor's case of neuralgia, 17.
Tendo Achillis, division of, 103.
Thompson Dr. John, death of, 360.
Thorn on sanguinaria, 397.
Tobacco in toothache, 7.
Toothache, 7, 314, 455.
Travis's case of concussion of the brain, 18.
Triplets, recognised by sounds, 221.

Umbilical cord, rupture of, 528.
University of Louisville, 176, 364.
" Virginia, 363.
Urnine, test for sugar in, 466.
Uteri cereix, excision of, 160.
Uterus, flexions and engorgements,65
Uterus inversion of, 16
Uterus, ulceration of neck of, 341.

Vaccination, 248.
Vagina, bloody vesicle of, 367.
" double, 81.
" rupture of, 532.
Veins varicose, 77.
Velpeau on flexions of uterus, 65.
" on inflammations, 225.
" on orchitis, 1.
Vesico-vaginal fistula, 470.
Vogel's Pathological Anatomy, 348.
Vomiting in pregnancy, mer. in, 167

Warren Prof. resignation of, 366.
Watson's introductory, 273.
Wendel's report of cases, 497.
Westcott's introductory, 274.
Western schools, 178.
White swelling, 172.
Wollaston the chemist, 170.
Worcester Prof. death of, 542.

Xylodine, 68.

Yandell D. W.'s letters from Paris, 1, 85, 93, 213, 308, 369, 400.
Yandell L. P.'s notice of Grayson Springs, 142.

